# Physiotherapy and optimised enteral nutrition in the post-acute phase of critical illness (PHOENIX): a randomised controlled feasibility trial

**DOI:** 10.1016/j.eclinm.2026.104000

**Published:** 2026-06-11

**Authors:** David McWilliams, Owen Gustafson, Nicola Wyer, Keith Couper, Peter K. Kimani, Rebecca Kandiyali, Asmaa El-Banna, Dalia Barghouthy, Rebekah Haylett, Elizabeth King, Holly Richardson, Miles Negus-Fancey, Louise Gallie, Violet Matthews, Zudin Puthucheary

**Affiliations:** a Centre for Care Excellence, Coventry University, Coventry, UK; b University Hospitals Coventry & Warwickshire NHS Trust, UK; c Oxford Allied Health Professions Research and Innovation Unit, Oxford University Hospitals NHS Foundation Trust, Oxford, UK; d Warwick Clinical Trials Unit, University of Warwick, Coventry, UK; e Critical Care Unit, University Hospitals Birmingham NHS Foundation Trust, Birmingham, UK; f Patient representative, UK; g William Harvey Research Institute, Queen Mary University of London, London, UK; h The Royal London Hospital, Barts Health NHS Trust, Whitechapel Road, London, UK

**Keywords:** Intensive care unit, Rehabilitation, Nutrition, Critical illness, Survivorship

## Abstract

**Background:**

Survivors of critical illness commonly experience prolonged physical and functional impairment related to muscle wasting, catabolism, and malnutrition. Rehabilitation and nutritional support beyond the intensive care unit (ICU) are frequently inconsistent, and deficits persist following ICU discharge. Although combined rehabilitation and nutritional interventions may support recovery, their feasibility in the post-ICU hospital setting is uncertain.

**Methods:**

PHOENIX was a mixed-methods, parallel group, open-label randomised controlled feasibility trial conducted in two United Kingdom university hospitals. Adult ICU survivors who had received four or more days of advanced respiratory support and with ongoing physiotherapy and dietetic rehabilitation needs at ICU discharge were recruited. Participants were randomly allocated to either an ICU-initiated, enhanced physiotherapy and optimised nutrition intervention or usual care. Primary feasibility outcomes were recruitment rate, retention at 30 days, and intervention fidelity. Secondary outcomes, collected to inform a future definitive trial, included days alive and out of hospital at 30 days (DAOH30), physical function, nutritional status, and quality of life. The trial was prospectively registered (ClinicalTrials.gov NCT06159868).

**Findings:**

Between May 2024 and January 2025, 172 patients were screened, of whom 60 met eligibility criteria and all consented to participate. Recruitment targets were achieved within eight months, and retention at 30 days was 100%. Enhanced physiotherapy was delivered on 81% of available days, exceeding the predefined feasibility threshold of 70%. Delivery of prescribed nutritional targets was feasible, with intake fidelity exceeding progression criteria for both calories (75.2%) and protein (83.7%).

**Interpretation:**

A combined enhanced physiotherapy and optimised nutrition intervention following ICU discharge is feasible, with high recruitment, retention, and intervention fidelity. A definitive multicentre randomised controlled trial is now warranted to evaluate clinical and cost-effectiveness.

**Funding:**

This project is funded by the 10.13039/501100000272National Institute for Health and Care Research (NIHR) under its Research for Patient Benefit (RfPB) Programme (Grant Reference Number NIHR205370).

**Research in context**

**Evidence before this study**

Survivors of critical illness frequently experience substantial muscle wasting, functional decline, and ongoing malnutrition after intensive care unit (ICU) discharge. Early rehabilitation and nutrition optimisation within the ICU have been widely studied, with mixed effects on long-term outcomes. However, comparatively little prospective randomised evidence addresses the post-ICU ward phase, where rehabilitation intensity often decreases and nutritional deficits persist. Prior studies have typically evaluated physiotherapy or nutrition in isolation, with limited data on combined multidisciplinary interventions, implementation fidelity, or feasibility of conducting large-scale trials in this setting.

**Added value of this study**

This randomised feasibility trial demonstrates that a combined enhanced physiotherapy and optimised nutrition intervention can be delivered after ICU discharge with high recruitment, retention, and intervention fidelity across two United Kingdom hospitals. The study provides detailed information on implementation processes, progression criteria, and health economic data collection to inform a definitive multicentre trial. It also highlights practical challenges in measuring total nutritional intake and anthropometric outcomes in the ward environment.

**Implications of all the available evidence**

Available evidence suggests that recovery after critical illness requires coordinated rehabilitation and nutritional strategies beyond the ICU. This study supports the feasibility of delivering and evaluating such a combined intervention and provides the methodological foundation for a larger trial to determine clinical and cost-effectiveness. If effective, this approach could inform structured post-ICU recovery pathways within hospital systems.

## Introduction

Over 200,000 adults are admitted to United Kingdom (UK) intensive care units (ICUs) each year, with more than 20 million globally.^1^^,^^2^ Approximately 80% survive, but up to two-thirds experience ongoing physical, psychological, and cognitive morbidity—collectively termed post-intensive care syndrome (PICS).^3^ One-third of working-age ICU survivors continue to require assistance with basic activities of daily living, around half are readmitted to hospital, and fewer than 50% return to work within one year.^4^^,^^5^ The associated economic burden is substantial, with secondary care costs in the year following ICU discharge estimated to approach £50,000 per patient.^6^ Consequently, national guidance from the National Institute for Health and Care Excellence (NICE) and the Intensive Care Society recommends early and structured rehabilitation throughout recovery.^7^^,^^8^ Despite this, the evidence base to support rehabilitation beyond the ICU remains limited.

Critical illness is characterised by a profound catabolic response, resulting in rapid skeletal muscle wasting, loss of strength, and impaired physical function. This process is driven by systemic inflammation, immobility, endocrine dysregulation, and nutritional inadequacy, and may continue well beyond the ICU stay.^9^^,^^10^ During ICU admission, patients commonly receive only around 60% of estimated energy and protein requirements because of feed interruptions, gastrointestinal intolerance, and procedural fasting.^11^^,^^12^ Consequently, large cumulative nutritional deficits accrue, often exceeding 18,000 kilocalories (kcal) and 1300 g (g) of protein per patient by the time of ICU discharge.^13^ These deficits contribute directly to sarcopenia, impaired rehabilitation potential, and delayed recovery.

The immediate post-ICU period represents a critical window for recovery. A national service evaluation across 25 UK centres found that, at ICU discharge, 98% of patients required ongoing physiotherapy and 70% were at risk of malnutrition.^14^ Despite this, ward-based rehabilitation is often inconsistent due to competing clinical priorities and suboptimal handover processes.^15^, ^16^, ^17^ Recent international survey data have further highlighted substantial variability in the delivery of post-ICU nutrition and rehabilitation in clinical practice, reinforcing the need for structured and coordinated approaches to support recovery.^18^ Existing evidence suggests that combining structured rehabilitation with optimised nutrition may be essential to support meaningful recovery following critical illness, but there is uncertainty as to the optimal approach^19^ and the feasibility of delivering these interventions. Randomised controlled trials and systematic reviews have demonstrated benefits of physical rehabilitation during ICU admission; however, evidence for interventions delivered following ICU discharge remains limited and heterogeneous, with few trials evaluating integrated rehabilitation and nutritional strategies and uncertainty regarding optimal timing, intensity, and delivery models.^20^^,^^21^

On this basis, we undertook the PHOENIX mixed methods feasibility randomised controlled trial (RCT) to assess the feasibility of undertaking a large clinical-effectiveness trial. Key objectives were to assess recruitment rates, intervention delivery, and follow-up.

## Methods

### Study design

PHOENIX was a mixed-methods, parallel-group randomised controlled feasibility trial conducted across two UK hospitals. Both sites were large university hospitals with established critical care and rehabilitation services. The present paper reports the quantitative feasibility and preliminary outcome findings. The qualitative component, which explored acceptability of the intervention and trial methodology, will be reported separately.

The trial was prospectively registered (ClinicalTrials.gov NCT06159868) and is reported in accordance with the CONSORT extension for pilot and feasibility trials.^22^ A detailed protocol has been published previously.^23^

### Ethics

The study was approved by the Wales Research Ethics Committee 2 (24/WA/0050). Written informed consent was obtained from participants or, where capacity was lacking, via an appropriate consultee.

### Participants

Eligible patients were adults (≥18 years) who had received ≥4 days advanced respiratory support (defined as invasive or non-invasive ventilation) and with on-going physiotherapy and dietetic rehabilitation needs. Ongoing rehabilitation needs were identified by the Post ICU presentation Screen (PICUPS) tool, defined as being unable to transfer from bed to chair independently and being unable to meet nutritional requirements independently. Exclusion criteria included poor pre-ICU admission mobility (inability to walk >10 m with or without an aid), contraindications to mobilisation or enteral nutrition, a significant acquired brain injury where the patient had not recovered to a Glascow Coma Scale (GCS) of ≥14 by ICU discharge, or where death was expected within the next 72 h.

All patients were screened daily by the critical care research team. For patients lacking capacity due to illness or sedation, a personal consultee or independent registered medical practitioner was approached. Participants were approached for consent once capacity was regained.

### Randomisation and masking

Permuted block randomisation was used to randomly allocate patients (1:1) to intervention or usual care using a secure electronic randomisation system (CASTOR) with allocation concealment, stratified by study site. Due to the nature of the intervention, blinding of participants and treating clinicians was not possible. Outcome assessments were performed by assessors blinded to group allocation.

### Procedures

#### Intervention

Participants allocated to the intervention received enhanced, individualised physiotherapy combined with optimised nutrition for up to 14 days following recruitment or until hospital discharge.

Enhanced physiotherapy was delivered by a dedicated physiotherapy-led team and included (1) Completion of a comprehensive physical and non-physical assessment to create an individualised treatment plan, (2) setting of patient centred rehabilitation goals with regular reassessment, (3) provision of daily physiotherapy (Monday-Friday), targeting the highest level of mobility attainable and delivery of individualised exercise programmes, (4) close working with ward staff to optimise treatment delivery and ensure seamless transitions of care once the enhanced intervention was complete.

Optimised nutrition was delivered by a specialist dietitian and involved comprehensive assessment of oral and enteral intake. Energy requirements were estimated using indirect calorimetry where feasible (COSMED QNRG+), or weight-based equations where not, with protein targets typically prescribed in the range of 1.2–2.0 g/kg/day in line with current clinical guidance. Individualised nutrition plans incorporated dietary modification, oral nutritional supplements and/or enteral feeding, with prescribed supplementation timed within 2 h of physiotherapy sessions to mitigate exercise-related catabolism. Nutrition plans were reviewed daily (Monday–Friday), with structured handover to ward teams following the intervention period.

A detailed description of the PHOENIX intervention in accordance with the Template for Intervention Description and Replication (TIDieR) checklist is provided in the Supplementary Table S1.

#### Usual care

Participants allocated to usual care received standard ward-based rehabilitation and dietetic care delivered during routine working hours, without additional input from ICU rehabilitation teams.

### Outcomes

Primary feasibility outcomes included 1) Recruitment and consent rates (overall and by centre); 2) retention rate (proportion of participants that complete the primary outcome); and 3) Intervention adherence (proportion of enhanced physiotherapy sessions completed and nutrition delivery compared to targets).

Secondary outcomes included measures that will be used in the future full-scale trial and were also collected at baseline, 14 days or hospital discharge (whichever came sooner), and at 30- and 90-days following randomisation. The proposed primary outcome for the definitive RCT will be days alive and out of hospital within 30 days (DAOH30). Out of hospital is classified as being at home or usual residence and defined as 30 minus the number of days in hospital (range 0–30), with 0 assigned for death within the 30 days. DAOH is a patient centred outcome, has been validated with a range of clinical specialities,^24^ and takes account those patients who die or are discharged to community rehabilitation settings.

Additional measures include 1) Physical function (30 second sit to stand test); 2) Functional independence (Barthel Index); 3) Quality of Life using the EuroQol 5 level EQ-5D (EQ-5D-5L); 4) DAOH at 90 days; 5) Hospital acquired malnutrition (Global Leadership Initiative on Malnutrition (GLIM) criteria); 6) Weight and percentage change in weight; 7)Time to therapy complete; 8) Hospital discharge destination; 9) Anthropometric measurements (mid arm circumference, triceps skin fold, hand grip strength).

#### Feasibility economic evaluation

The focus for the economic feasibility study was on optimising the quality of data collected for the first 90 days post-randomisation.

Data collection included: (1) resource use data on the components of the intervention, including physiotherapist and dietitian staff time; use of rehabilitation aids and dietary interventions to provide a preliminary estimate of the costs associated with delivering the PHOENIX intervention and (2) patient-level resource use within the broader healthcare system (e.g. outpatient, GP and other NHS healthcare professional appointments) at 30 and 90 days post randomisation to assess the feasibility of data collection.

Details relating to the intervention were captured by case report forms completed prospectively. Subsequent healthcare resource use, including primary care and other services not covered within the case report forms (CRFs) were captured via the validated Modular resource-use measure (MODRUM).^25^

### Statistical analysis

The sample size of 60 participants was selected to allow robust estimation of feasibility outcomes including recruitment, retention, and intervention fidelity, and to inform progression criteria for a definitive trial. Based on prespecified thresholds, this sample size provided a high probability of correctly identifying whether progression criteria were met across key feasibility domains. Randomisation was stratified by study site to ensure balance between groups. As this was a feasibility study, the analysis was primarily descriptive and not designed to formally estimate treatment effects or support inferential comparisons. Given the small sample size and the limited number of sites (n = 2), formal adjustment or subgroup analyses by site were not performed, as these would be underpowered and risk over-interpretation.

Descriptive analysis was used to summarise patient demographics and other characteristics at baseline. Raw frequencies and percentages were used for categorical characteristics while mean (standard deviation [SD]), median (interquartile range [IQR]), were used for continuous and ordinal data. The summaries were computed for all patients and for the usual care group and the enhanced physiotherapy and optimised nutrition group. Missing data were assumed to be missing at random, and analyses were conducted using available case analysis given the feasibility nature of the study. Continuous variables were assessed for normality using visual inspection of histograms and summary statistics.

As data were approximately normally distributed, they are presented as mean (SD) and comparisons between groups were made using independent t-tests. Categorical variables were compared using Fisher's exact test. All analyses were performed using IBM SPSS Statistics version 22 (IBM Corp., Armonk, NY). A traffic light ‘stop-amend-go’ system^26^ was established *a priori* to guide decision-making for a definitive trial (See Supplementary Table S2). Recruitment rate was defined as the percentage of the target sample recruited during the feasibility trial. Retention was defined as the proportion of patients where it was possible to collect their days alive out of hospital at 30 days (DAOH30).

For physiotherapy feasibility, overall fidelity was the percentage of days that physiotherapy was provided to all patients in each arm during the intervention period. To complement this, fidelity was also evaluated at an individual patient level. Nutrition feasibility was assessed using calories and protein intake separately. Proportions were calculated for the actual calories and protein amounts consumed, against the sum of daily targets for prescribed oral nutritional supplements and/or enteral tube feeds during the intervention period. Again, these were evaluated at a group and individual level. Calorie and protein intakes were capped so that if intake was higher than the target, it was set to be equal to the calorie and protein target.

DAOH30 scores were assessed to inform feasibility of data collection, distributional properties, and potential analytical approaches for a future definitive trial. Secondary outcomes were analysed descriptively to explore data completeness, distribution, and potential signals to inform the design of a future trial. No formal hypothesis testing or adjustment for multiple comparisons was undertaken.

### Role of the funding source

This project is funded by the National Institute for Health and Care Research (NIHR) under its Research for Patient Benefit (RfPB) Programme (Grant Reference Number NIHR205370). The funder had no role in study design, data collection, data analysis, data interpretation, or writing of the report. The views expressed are those of the authors and not necessarily those of the NIHR or the Department of Health and Social Care.

## Results

### Recruitment feasibility

**Fig. 1:**
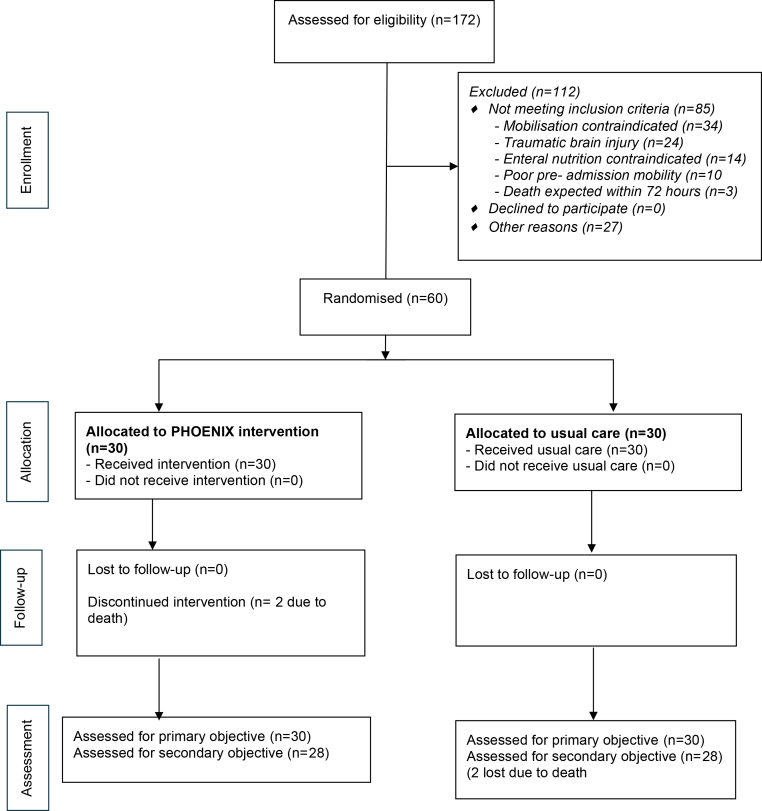
CONSORT pilot and feasibility trials flow schematic.

A total of 172 patients were screened across the two hospitals during the eight-month recruitment period (from 7th May, 2024 to 8th January 2025). Of these, 60/172 (35%) met the inclusion criteria and were invited to participate; and 60/60 (100%) consented and were randomly allocated to either the enhanced physiotherapy and optimised nutrition or the usual care group (see Fig. 1). The target recruitment rate of 60 patients was achieved within an eight-month time frame. The most common reasons for ineligibility were contraindications to mobilisation (34/112, 30%), traumatic brain injury with a GCS ≤14 at screening (24/112, 21%) and contraindications to enteral nutrition (10/112, 9%).

### Sample characteristics

Participants were mostly white (54/60; 90%), male (39/60; 65%), with a mean age of 60.8 years. The most common primary diagnosis on admission to ICU were respiratory (36.7%), surgery (18.3%) or trauma (16.7%). Groups were balanced at baseline (see Table 1).

**Table 1:** Demographics and baseline characteristics.

Characteristic	All patients (n = 60)	Usual care (n = 30)	Intervention (n = 30)
Sex, n (%) Male	39 (65.0)	19 (63.3)	20 (66.6)
Age			
Mean (SD)	60.8 (15.2)	60.2 (15.2)	61.4 (15.4)
Median (IQR)	64.5 (49.0, 74.0)	65.0 (52.0, 70.2)	63.5 (49.0, 75.0)
Ethnicity, n (%)			
White–British	50 (83.3)	23 (76.7)	27 (90.0)
White–other white	4 (6.7)	3 (10.0)	1 (3.3)
Asian/Asian British–Indian	5 (8.3)	3 (10.0)	2 (6.7)
Other	1 (1.7)	1 (3.3)	0 (0.0)
Functional comorbidity index			
0	17 (28.3)	10 (33.3)	7 (23.3)
1	19 (31.7)	6 (20.0)	13 (43.3)
2	11 (18.3)	9 (30.0)	2 (6.7)
3–7	13 (21.7)	5 (16.7)	8 (26.7)
APACHE II score	Missing cases (n = 7)	Missing cases (n = 3)	Missing cases (n = 4)
Mean (SD)	18.2 (5.6)	18.2 (6.2)	18.3 (5.0)
Median (IQR)	18.0 (14.0, 21.0)	18.0 (13.0, 22.5)	18.0 (16.0, 20.8)
Primary diagnosis			
Respiratory	22 (36.7)	9 (30.0)	13 (43.3)
Surgery	11 (18.3)	4 (13.3)	7 (23.3)
Trauma	10 (16.7)	6 (20.0)	4 (13.3)
Neuro	6 (10.0)	3 (10.0)	3 (10.0)
Cardiovascular	5 (8.3)	4 (13.3)	1 (3.3)
Sepsis	3 (5.0)	3 (10.0)	0 (0.0)
Other	3 (5.0)	1 (3.3)	2 (6.7)
ICU length of stay (days)			
Mean (SD)	18.0 (14.2)	17.9 (14.1)	18.0 (14.4)
Median (IQR)	14.5 (10.0, 19.0)	14.0 (10.0, 19.5)	15.0 (10.0, 19.0)
Sedated, n (%) Yes	55 (91.7)	27 (90.0)	28 (93.3)
Sedated days			
Mean (SD)	8.3 (5.8)	8.3 (6.5)	8.2 (5.0)
Median (IQR)	7.0 (4.0, 11.0)	7.5 (4.0, 11.0)	7.0 (5.0, 11.0)
Ventilation, n (%)			
Non-invasive only	3 (5.0)	2 (6.7)	1 (3.3)
Invasive only	36 (60.0)	19 (63.3)	17 (56.7)
Invasive and non-invasive	21 (35.0)	9 (30.0)	12 (40.0)
Advanced support days			
Mean (SD)	15.0 (13.9)	14.3 (13.7)	15.6 (14.3)
Median (IQR)	11.0 (7.8, 16.2)	11.0 (7.0, 13.8)	12.0 (8.0, 18.5)

SD = Standard deviation, IQR = Interquartile range, APACHE II = Acute Physiology and Chronic Health Evaluation II.

At baseline 48/60 (80%) of patients were unable to perform any stands during the 30 s sit to stand (STS), 14/60 (23.3%) were unable to perform any activities of daily living based on the Barthel Index, although these were split equally between groups (See Table 2). Patients in the intervention group had lost more weight during their ICU stay (mean loss 6.8 vs 3.4 kg) and were more likely to have malnutrition at baseline assessment based on the GLIM criteria (97% vs 63%).

**Table 2:** Baseline characteristics at ICU discharge.

Characteristic	All patients (n = 60)	Usual care (n = 30)	Intervention (n = 30)
30 Second STS, n (%)			
0	48 (80.0)	24 (80.0)	24 (80.0)
1	3 (5.0)	1 (3.3)	2 (6.7)
2	3 (5.0)	0 (0.0)	3 (10.0)
3	4 (6.7)	4 (13.3)	0 (0.0)
4	1 (1.7)	1 (3.3)	0 (0.0)
5	1 (1.7)	0 (0.0)	1 (3.3)
Barthel index, n (%)			
0	14 (23.3)	7 (23.3)	7 (23.3)
5	17 (28.3)	10 (33.3)	7 (23.3)
10	6 (10.0)	3 (10.0)	3 (10.0)
15	7 (11.7)	3 (10.0)	4 (13.3)
20–70	16 (26.7)	7 (23.3)	9 (30.0)
Manchester mobility score			
Median (IQR)	4 (3, 5)	4 (3,5)	4 (4,5)
Weight, kg			
Mean (SD)	79.5 (18.5)	81.7 (19.8)	77.4 (17.2)
Median (IQR)	78.5 (67.8, 90.0)	82.6 (70.2, 89.3)	76.0 (65.0, 89.7)
BMI, kg/m2			
Mean (SD)	27.0 (6.3)	27.9 (7.3)	26.1 (5.1)
Median (IQR)	26.5 (23.6, 29.6)	26.7 (24.7, 30.6)	26.1 (22.3, 28.1)
Weight change in ICU	Missing cases = 5	Missing cases = 4	Missing cases = 1
Mean (SD)	−5.2 (7.7)	−3.4 (9.7)	−6.8 (5.1)
Median (IQR)	−5.4 (−9.1, −1.7)	−1.8 (−5.5, 1.9)	−6.3 (−9.7, −3.5)
Mid upper arm circumference	Missing cases = 1	Missing cases = 1	
Mean (SD)	30.0 (5.2)	30.3 (6.2)	29.7 (4.2)
Median (IQR)	29.0 (26.8, 32.0)	29.0 (26.0, 33.0)	29.2 (28.0, 31.0)
Tricep skinfold thickness	Missing cases = 1	Missing cases = 1	
Mean (SD)	13.7 (7.8)	14.9 (9.9)	12.6 (5.0)
Median (IQR)	13.0 (9.5, 16.0)	12.0 (10.0, 16.5)	13.1 (9.2, 16.0)
Mid arm muscle circumference	Missing cases = 1	Missing cases = 1	
Mean (SD)	25.6 (4.1)	25.6 (4.4)	25.6 (3.9)
Median (IQR)	25.3 (23.0, 27.6)	25.4 (22.7, 27.9)	25.2 (23.3, 26.8)
GLIM, n (%)			
No Malnutrition	12 (20.0)	11 (36.7)	1 (3.3)
Moderate malnutrition	44 (73.3)	16 (53.3)	28 (93.3)
Severe malnutrition	4 (6.7)	3 (10.0)	1 (3.3)

SD = Standard deviation, IQR = Interquartile range, STS = Sit to stand, kg = Kilogrammes, BMI = Body Mass Index, Glim = Global Leadership Initiative on Malnutrition.

### Retention

Fig. 1 shows the flow of participants through the study, including those lost to follow up over 30 days. DAOH30 score (the proposed primary outcome in a future definitive trial) was collected for all patients, although following recruitment 4 participants died (2 from each group) meaning secondary outcomes were only available for 28 patients in each group.

### Intervention fidelity

Patients in the intervention arm received significantly more physiotherapy and dietetic input during the intervention period (See Table 3). This was associated with significant increase in the completion of assessments and goal setting.

**Table 3:** Rehabilitation provision and fidelity.

	Usual care	Intervention	p-value
Intervention fidelity % – Median (IQR)			
Physiotherapy	64 (44.6, 71.4)	84 (71.4, 100.0)	
Calories	68.6 (28.7, 100.0)	75.2 (62.2, 99.3)	
Protein	66.1 (29.5, 99.6)	83.7 (62.6, 93.1)	
Physiotherapy			
Number of contacts	6.53 (5.02)	11.67 (6.93)	0.002
Duration (mins)	179.67 (132.29)	367.00 (185.77)	<0.001
Nutrition contacts			
Dietitian	1.77 (1.14)	4.17 (3.80)	0.002
Assistant practitioner	0.13 (0.43)	0.83 (1.49)	0.017
Comprehensive assessment			
Baseline	14/30 (47%)	29/30 (97%)	<0.001
Week 1	0/21 (0%)	19/22 (86%)	<0.001
Week 2	0/21 (0%)	11/16 (69%)	<0.001
Goals set/updated			
Baseline	14/30 (47%)	29/30 (97%)	<0.001
Week 1	2/21 (10%)	19/22 (86%)	<0.001
Week 2	1/21 (5%)	12/15 (80%)	<0.001
Rehab plan			
Baseline	14/30 (47%)	29/30 (97%)	<0.001
Week 1	1/21 (5%)	19/22 (86%)	<0.001
Week 2	0/21 (0%)	12/15 (80%)	<0.001
Nutritional plan updated			
Week 1	14/21 (67%)	20/21 (95%)	0.045∗
Week 2	9/20 (45%)	13/14 (93%)	0.009

IQR = Interquartile range.

In total, enhanced physiotherapy was provided in 240 of 298 potential opportunities for participants in the intervention group, giving an overall physiotherapy fidelity rate of 81%. This exceeded the predefined fidelity threshold of 70%, Similarly, intervention fidelity was also demonstrated for the delivery of both calories (361,993.5/507,483; 71.3%) and protein (17,962.1/23,017.9; 78%).

The intensity of physiotherapy was individualised, with patients supported to achieve the highest level of mobility appropriate to their clinical status, progressing from bed–based activity to sitting, standing, and ambulation where feasible. The mean (SD) duration of physiotherapy delivered in the intervention group was 367 (185.77) minutes over the intervention period. Rehabilitation goals were individualised and regularly reviewed, with a high proportion of patients in the intervention group having documented goals and rehabilitation plans established and updated throughout the intervention period (Table 3). Further details on the individual patient change in mobilisation levels is provided in Supplementary Table S3.

#### Secondary outcomes

All secondary outcome analyses were exploratory and intended to inform outcome selection and trial design rather than to assess intervention effectiveness.

#### DAOH30 outcome data

By day 30, 45 patients had been discharged from hospital with four patients having died (two in each group) and 11 remaining in hospital (five in usual care group; six patients in the enhanced physiotherapy and optimised nutrition group). The median (IQR) DAOH30 scores for the enhanced physiotherapy and optimised nutrition and the usual care groups were 16 (0.5, 23.75) and 13 (3.75, 24.75) respectively.

### DAOH 90 days

Within ninety days, there were six deaths, two in the usual care group and four in the intervention group. Further, two patients in the usual care group and one patient in the intervention group stayed in hospital more than 90 days so that their DAOH90 scores were zero. The median (IQR) DAOH90 scores for the enhanced physiotherapy and optimised nutrition and the usual care groups were 75 (56.00, 82.75) and 73 (61.50, 85.00) respectively.

### Ward length of stay

Median (IQR) ward length of stay was 12.5 (6.0–21.25) days in the intervention group and 17.0 (5.0–23.25) days in the usual care group. These findings are exploratory and not powered for formal between-group comparison.

### EQ-5D-5L

Mean utility scores were consistently higher in the PHOENIX intervention group at all time points. At 14 days, mean utility scores were 0.467 (95% CI 0.316–0.619) in the intervention group and 0.446 (95% CI 0.233–0.658) in the usual care group. At 30 days, mean utility scores were 0.564 (95% CI 0.369–0.759) in the intervention group and 0.487 (95% CI 0.265–0.708) in the usual care group. However, the substantial overlap in confidence intervals indicates considerable uncertainty around the estimates, reflecting the small sample size.

### Physical outcomes

Patients in the intervention group had higher mean 30 second STS scores at both 14 days (3.1 vs 1.7) and 30 days (3.7 vs 2.4), with a greater proportion of patients demonstrating improvement in the intervention group compared with usual care (26.1% vs 59.3%, p = 0.019). Intervention patients demonstrated higher median (IQR) changes in Barthel scores in comparison to the usual care group at both 14 days (50, 45–90 vs 35, 15–95), and 30 days (70, 60–90 vs 60, 40–85). A greater proportion of intervention patients were able to walk at least 30 m at hospital discharge compared with usual care (71.4% vs 37.9%, p = 0.009).

### Weight (14 and 30 days)

Patients in the intervention arm lost less weight during the intervention period (mean ± SD, −0.56 ± 4.73 vs −1.85 ± 6.31 kg) although overall weight loss was similar at 30 days (−3.02 ± 9.40 vs −2.62 ± 8.65 kg). A larger proportion of patients in the usual care group met GLIM criteria for malnutrition at hospital discharge compared with the intervention group (9/29, 31% vs 1/28, 3.6%).

### Mid arm muscle circumference

Mid arm muscle circumference (MAMC) for both baseline and at 30 days were obtained from 11 patients in each group. On average, small numerical increases in mid-arm muscle circumference were observed in the intervention group, while small decreases were observed in the usual care group (mean ± SD, 0.39 ± 3.26 vs −0.83 ± 2.65 p = 0.588), but the number of available patient data for this measure was small, and the changes not statistically different.

### Handgrip strength

Handgrip strength statistically increased in both intervention and usual care groups at both 14 (mean ± SD, 4.33 ± 4.51 versus 3.36 ± 4.50) and 30 days (7.99 ± 4.90 versus 8.61 ± 9.05), with no significant difference between the groups.

### Health economics

The PHOENIX intervention increased in-hospital resource use and costs compared with usual care (mean ± SD, £904.86 ± £418.33 versus £175.10 ± £101.36) Table 4 presents a breakdown of the physiotherapy and nutrition-related cost elements for each study arm. Increased in-hospital resource use in the intervention group was primarily driven by additional physiotherapy and dietetic staff time, close working with ward staff for the first 10 days of the intervention and the provision of an optimised nutrition plan. Although the intervention was associated with increased in-hospital staff costs, exploratory differences in ward length of stay suggest that downstream cost offsets should be formally evaluated in a definitive trial.

**Table 4:** Intervention per patient costs.

	Usual care	Intervention
Physiotherapy cost (£)		
Mean ± SD	£125.77 ± £92.60	£420.70 ± £185.15
Min – Max	£10.50 – £273.00	£49.00 – £717.50
Nutrition cost (£)		
Mean ± SD	£38.83 ± £24.08	£473.66 ± £241.93
Min – Max	£0.00 – £105.00	£0.00 – £783.15

SD = standard deviation, Min = minimum, Max = maximum.

The completion rates of the ModRUM questionnaire at 30 and 90 days were analysed to identify any issues or barriers to completion. The completion rate was 62% at 30 days and 47% at 90 days. The main reason for non-completion at 30 days was that patients remained as inpatients at this time point. At 90 days non completion was primarily due to patients falling outside the 90-day window following a protocol update.

## Discussion

This feasibility trial, embedded within a mixed-methods programme, demonstrates that a combined enhanced physiotherapy and optimised nutrition intervention initiated at ICU discharge can be delivered with high recruitment, retention, and intervention fidelity. The high consent and retention rates likely reflect the timing of approach during a period of high patient engagement, the perceived relevance of the intervention to recovery, and close integration with clinical care pathways. Progression criteria were met across all primary feasibility domains, supporting advancement to a definitive multicentre randomised controlled trial.

Although not powered to assess effectiveness, exploratory outcomes provide useful signals to inform future trial design. Numerically greater improvements were observed across several physical and functional measures, alongside higher days alive and out of hospital, in the intervention group. These findings are strictly hypothesis-generating and should not be interpreted as evidence of efficacy. However, they support the feasibility and suitability of candidate outcome measures and reinforce the appropriateness of days alive and out of hospital as a patient-centred primary endpoint in the post-ICU setting.

A key strength was the high fidelity of intervention delivery. Enhanced physiotherapy was provided on more than 80% of available days, exceeding predefined thresholds and substantially greater than exposure under usual care. Nutritional targets for both energy and protein were achieved above progression criteria. Importantly, this was accomplished despite greater baseline weight loss and a higher prevalence of malnutrition in the intervention group. While this baseline imbalance reflects random variation within a small feasibility sample and complicates interpretation of exploratory outcomes, it also demonstrates that the intervention can be delivered reliably even among patients at highest nutritional risk. In a definitive trial, stratification by baseline nutritional status and prespecified covariate adjustment (e.g. weight loss and malnutrition risk) will be used to address such imbalances.

The observed differences in nutritional delivery between groups appear to be driven by several factors inherent to the intervention design. Comprehensive dietetic assessment and daily review enabled recalibration of nutritional targets appropriate to the recovery phase, alongside timely adjustments to improve tolerance and acceptability. Aligning nutritional supplementation with physiotherapy sessions was intended to mitigate exercise-induced catabolism and reflects physiological principles underpinning muscle protein synthesis. While this study was not designed to test mechanistic hypotheses, these processes warrant further investigation in future trials incorporating appropriate biological and functional endpoints.

Consistent with previous literature,^16^^,^^27^ usual care following ICU discharge remained variable, with inconsistent physiotherapy exposure and fragmented nutritional support. Despite receiving higher levels of rehabilitation than reported in some earlier cohorts, patients allocated to usual care did not receive daily physiotherapy, and a substantial proportion developed new or worsening malnutrition during the ward-based recovery phase. These findings reinforce the ICU-to-ward transition as a modifiable gap in recovery pathways and support the rationale for structured, multidisciplinary post-ICU interventions.

This feasibility trial also provided important insights into the conduct of economic evaluation alongside a future definitive study. While the intervention increased in-hospital costs driven primarily by staff time and nutritional provision, the study was not designed to capture potential downstream cost offsets such as reduced length of stay, readmissions, or community healthcare utilisation. Future research should therefore ensure comprehensive collection of patient-level resource use, including staff contacts, nutritional products, rehabilitation aids, and post-discharge healthcare utilisation.

A definitive trial will include a full economic evaluation conducted from an NHS and personal and social services perspective, reporting incremental costs, QALYs and net monetary benefit derived from EQ-5D-5L, likely using a lifetime horizon. Such evaluation will determine whether early investment in coordinated rehabilitation and nutritional support during the post-ICU recovery phase can translate into improved outcomes and downstream reductions in healthcare utilisation.

Several methodological refinements occurred between the published protocol and trial conduct, to optimise feasibility processes and inform the design of the future definitive multicentre trial. Acceptability, originally described as a primary outcome assessed through qualitative methods, is reported separately to allow appropriate methodological depth; feasibility progression criteria in this manuscript therefore focus on recruitment, retention, and intervention fidelity.

Definitions of intervention fidelity were pragmatically adapted. Nutritional fidelity was reported as the proportion of estimated energy and protein requirements achieved, rather than solely the proportion of prescribed supplements consumed, to better reflect total delivered intake. Physiotherapy fidelity was calculated per available day rather than per session to account for ward-based scheduling variability. These refinements provide a more clinically meaningful estimate of intervention exposure and will inform the definitive protocol.

Although daily capture of total nutritional intake was specified, oral dietary intake from food sources was not consistently recorded due to ward documentation limitations. The definitive trial will incorporate structured dietary recording processes to improve completeness. Some prespecified exploratory outcomes, including return to work, were not reported due to limited short-term events and will be incorporated into longer-term follow-up in the multicentre study.

This study has several limitations that should be considered when interpreting the findings. Firstly, the included cohort reflects a typical population of ICU survivors requiring prolonged respiratory support and ongoing rehabilitation needs, although eligibility criteria and exclusion of patients with severe neurological impairment may limit generalisability to all ICU populations. Dietary intake from food sources was not reliably captured, limiting assessment of total energy and protein intake. Indirect calorimetry was feasible in only a small subset of participants due to equipment availability and clinical constraints. Outcome measurements were scheduled for 14 days or at hospital discharge, whichever occurred first, resulting in variability in the timing of data collection. Anthropometric measurements such as mid arm muscle circumference were available for only a subset of patients, reflecting the practical challenges of data collection in this population, including early discharge and patient-related or clinical constraints. As with most rehabilitation trials, blinding of participants and treating clinicians was not possible, although outcome assessors were masked to allocation. The proportion of male participants in this study (65%) is broadly consistent with national UK critical care admission data, where approximately 57.5% of admissions are male, suggesting the study cohort is representative of the wider ICU population. Nevertheless, future multicentre trials should continue to monitor recruitment patterns to ensure representation across sex and other demographic characteristics. Finally, exploratory outcome analyses were underpowered and should not be interpreted as evidence of effectiveness.

Overall, this feasibility trial demonstrates that a combined enhanced physiotherapy and optimised nutrition intervention following ICU discharge can be delivered with high fidelity and patient engagement in routine ward practice. Recruitment, retention, and outcome collection processes support progression to a definitive multicentre trial. A fully powered randomised controlled study with embedded economic evaluation is now required to determine the clinical and cost-effectiveness of this integrated recovery intervention in survivors of critical illness.

## Contributors

DM and ZP contributed to the conceptualisation of the research. DM, ZP, NW, and OG developed the intervention. DM, ZP, OG, KC, NW, PKK, RK, MNF, LG, and VM contributed to the study design and development of the study protocol. VM was the trial manager and oversaw trial delivery and coordination. EK, MNF, and LG led the patient and public involvement activities for the development of the protocol. RH, DB, and HR were responsible for participant recruitment and data collection. DM, NW, RK, AE-B, and PKK directly accessed and verified the underlying data. OG and DM led drafting of the manuscript. All authors critically reviewed the manuscript, provided intellectual input, and approved the final version for submission.

## Data sharing statement

Individual participant data underlying the results reported in this article will not be made publicly available because consent for wider data sharing was not obtained from participants at the time of recruitment.

## Declaration of interests

Zudin Puthucheary is a trustee for the Intenisve Care Society and the National Confidential Enquiry into Patient Outcomes and Death (NCEPOD). He has received grants, consultancy fees, and Honoria payments for lectures from Nestle Health Sciences and grants and consultancy fees from Fresenius Kabi, He is also a named inventor on a ketogenic feed.

Keith Couper is NIHR Research for Patient Benefit funding panel chair for the West Midlands.

Owen Gustafson is funded by the National Institute for Health and Care Research (NIHR) Oxford Biomedical Research Centre (BRC). The views expressed are those of the author(s) and not necessarily those of the NIHR or the Department of Health and Social Care.

Elizabeth King is Journal co-editor for the Association of Chartered Physiotherapists in Respiratory Care (ACPRC).

The authors have no other conflicts of interest to declare.

## Appendix A Supplementary data

Supplementary Material

PHOENIX trial changes summary

Protocol
