# Supplementary material for: Physiotherapy and optimised enteral nutrition in the post-acute phase of critical illness (PHOENIX): a randomised controlled feasibility trial
Source: eClinicalMedicine. 2026 Jun 11;96:104000. doi: 10.1016/j.eclinm.2026.104000 (PMC13276347; doi:10.1016/j.eclinm.2026.104000)
Supplement: Supplementary Material [file mmc1.docx]

**Supplement**

**Supplementary Table 1. Description of the PHOENIX intervention according to the Template for Intervention Description and Replication (TIDieR) checklist**

| **TIDieR Item** | **Description of PHOENIX Intervention** |
| --- | --- |
| **1. Brief name** | **PHOENIX intervention:** A combined programme of enhanced physiotherapy and optimised nutrition delivered following ICU discharge to support recovery from critical illness. |
| **2. Why (Rationale)** | Survivors of critical illness commonly experience muscle wasting, functional decline, and persistent malnutrition. Muscle recovery requires both **exercise stimulus and adequate nutritional substrate**. The PHOENIX intervention was designed to integrate physiotherapy and nutritional optimisation during the early post-ICU ward phase, when rehabilitation needs are high but therapy provision is often inconsistent. |
| **3. What (Materials)** | Standard clinical resources were used, including physiotherapy rehabilitation equipment (mobility aids, resistance bands, functional exercise tools), nutritional assessment tools, indirect calorimetry equipment (where available), oral nutritional supplements, modified hospital diets, and enteral feeding regimens. Individualised physiotherapy and nutrition care plans were documented within patient records. |
| **4. What (Procedures)** | **Physiotherapy:** Comprehensive assessment; development of individualised rehabilitation plan; patient-centred goal setting; daily physiotherapy sessions targeting mobility, strength, endurance, and functional activity; regular reassessment and progression of rehabilitation goals; coordination with ward staff to support activity outside therapy sessions.   **Nutrition:** Comprehensive nutritional assessment; determination of energy requirements using indirect calorimetry where feasible (otherwise predictive equations); calculation of protein targets using weight-based equations; development of an individualised nutrition plan including dietary modification, enhanced food provision, oral nutritional supplements, and/or adjustment of enteral feeding regimens. Where possible, a nutritional supplement dose was scheduled within **two hours of physiotherapy sessions** to support anabolic recovery. |
| **5. Who provided** | Specialist rehabilitation team consisting of **physiotherapists experienced in critical care rehabilitation** and **dietitians experienced in nutrition support and critical illness recovery**, working collaboratively and liaising with ward multidisciplinary teams. |
| **6. How (Mode of delivery)** | Delivered **face-to-face on hospital wards** following ICU discharge. The specialist physiotherapy and dietetic teams provided the intervention while coordinating with usual ward staff to optimise continuity of care. |
| **7. Where** | General hospital wards in **two UK university hospitals** following patient discharge from intensive care. |
| **8. When and how much** | Intervention delivered for **up to 14 days following ICU discharge or until hospital discharge**, whichever occurred sooner. Physiotherapy sessions were provided **daily Monday–Friday**. Dietetic review and adjustment of nutrition plans also occurred **daily Monday–Friday** during the intervention period. |
| **9. Tailoring** | Both physiotherapy and nutritional components were **individualised according to patient clinical status, functional ability, and nutritional requirements**. Rehabilitation progression and nutritional prescriptions were adjusted according to patient response and recovery trajectory. |
| **10. Modifications** | No major protocol modifications were made during the feasibility trial. Routine adjustments to rehabilitation and nutrition plans occurred as part of individualised patient care. |
| **11. Planned fidelity assessment** | Intervention fidelity was assessed prospectively. **Physiotherapy fidelity:** proportion of eligible days on which enhanced physiotherapy was delivered. **Nutrition fidelity:** proportion of prescribed calorie and protein targets achieved. |
| **12. Actual fidelity** | Enhanced physiotherapy was delivered on **81% of eligible days**, exceeding the predefined feasibility threshold (70%). Mean nutritional intake fidelity exceeded progression criteria for both **calories (75.2%)** and **protein (83.7%)**, demonstrating feasibility of delivering the intervention in routine ward care. |

**Supplementary** **Table 2: Traffic light system to assess feasibility of a future definitive randomised controlled trial.**

|  | Green  (Trial feasible) | Amber  (May be feasible with modification) | Red  (Likely unfeasible) |
| --- | --- | --- | --- |
| Recruitment of proposed rate (%) | 100%  (60 participants) | 60- 99%  (36-59 participants) | <60%  (<36 participants) |
| Retention rates – defined as collection of primary outcome measure (days alive and out of hospital) | 100%  (60 participants) | 60 - 99%  (36-59 participants) | <50%  (<36 participants) |
| Intervention fidelity (Physiotherapy)  Proportion of available sessions completed | > 70% | 40 – 70% | <40% |
| Intervention fidelity (nutrition)  Proportion of prescribed supplements (oral / enteral tube feed) taken | >70% | 40-70% | < 40% |

**Supplementary** **Table 3: Cross tabulation of Manchester Mobility Scores at baseline and at 14 days for usual care and intervention groups**

| **Score at baseline** | **Usual care (n=29)** | | | | | | | |  | **Intervention (n=28)** | | | | | | | |
| --- | --- | --- | --- | --- | --- | --- | --- | --- | --- | --- | --- | --- | --- | --- | --- | --- | --- |
|  | **Score at 14 days†** | | | | | | | |  | **Score at 14 days†** | | | | | | | |
|  | **A** | **B** | **C** | **D** | **E** | **F** | **G** | **Total, n (%)** |  | **A** | **B** | **C** | **D** | **E** | **F** | **G** | **Total, n (%)** |
| In bed interventions only (**A**) | 0 | 0 | 0 | 0 | 0 | 0 | 0 | 0 (0) |  | 0 | 0 | 0 | 1 | 1 | 0 | 2 | 4 (14.3) |
| Sit on edge of bed (**B**) | 0 | 2 | 1 | 1 | 0 | 1 | 3 | 8 (27.6) |  | 0 | 0 | 0 | 1 | 0 | 0 | 1 | 2 (7.1) |
| Hoisted to chair (including standard hoist) (**C**) | 0 | 0 | 0 | 1 | 0 | 2 | 0 | 3 (10.3) |  | 0 | 0 | 0 | 1 | 0 | 0 | 0 | 1 (3.6) |
| Standing practice (**D**) | 0 | 1 | 1 | 2 | 1 | 0 | 0 | 5 (17.2) |  | 0 | 0 | 0 | 1 | 0 | 3 | 6 | 10 (35.7) |
| Step transfers with assistance (**E**) | 0 | 0 | 0 | 0 | 3 | 1 | 6 | 10 (34.5) |  | 0 | 0 | 0 | 0 | 0 | 0 | 8 | 8 (28.6) |
| Mobilising with or without assistance (**F**) | 0 | 0 | 0 | 0 | 0 | 1 | 2 | 3 (10.3) |  | 0 | 0 | 0 | 0 | 0 | 0 | 3 | 3 (10.7) |
| Mobilising >30 metres with or without assistance (**G**) | 0 | 0 | 0 | 0 | 0 | 0 | 0 | 0 (0) |  | 0 | 0 | 0 | 0 | 0 | 0 | 0 | 0 (0) |
| **Total, n (%)** | 0  (0) | 3  (10.3) | 2  (6.9) | 4  (13.8) | 4  (13.8) | 5  (17.2) | 11  (37.9) |  |  | 0  (0) | 0  (0) | 0  (0) | 4  (14.3) | 1  (3.6) | 3  (10.7) | 20  (71.4) |  |
| **Median (LQ, UQ)** | 6.0 (4.0, 7.0) | | | | | | |  |  | 7.0 (6.0, 7.0) | | | | | | |  |
|  | † The scores corresponding to different letters are given in the first column | | | | | | | | | | | | | | | | |
|  |  | Worse score at 14 days | | | |  | Same score | |  |  | Improve by 1 unit | | |  | Improve by ≥ 2 units | | |
