# Supplementary material for: Physiotherapy and optimised enteral nutrition in the post-acute phase of critical illness (PHOENIX): a randomised controlled feasibility trial
Source: eClinicalMedicine. 2026 Jun 11;96:104000. doi: 10.1016/j.eclinm.2026.104000 (PMC13276347; doi:10.1016/j.eclinm.2026.104000)
Supplement: PHOENIX trial changes summary [file mmc2.docx]

**PHOENIX Trial – Summary of Amendments from Published Protocol**

To summarise, the PHOENIX feasibility trial was conducted in accordance with the published protocol (BMJ Open 2025;15:e100803). No substantive changes were made to the study design, eligibility criteria, or intervention. For transparency, the following clarifications and minor amendments should be noted:

**Study reporting**

The qualitative component specified in the protocol was undertaken as planned; qualitative findings are to be reported separately. The submitted manuscript focuses on quantitative feasibility and exploratory outcomes.

**Feasibility outcomes**

Retention was defined as completion of the primary outcome (days alive and out of hospital at 30 days [DAOH30]). Terminology was standardised in the manuscript, with “intervention adherence” used in place of “intervention fidelity”. In addition, some prespecified exploratory outcomes (e.g. return to work or usual activity at 30 and 90 days) are not reported in the present manuscript and will be reported separately or were not available for complete analysis at this stage.

**Intervention fidelity**

Operational definitions of fidelity were prespecified in the protocol and further refined in the analysis. Physiotherapy fidelity was defined as the proportion of available days on which therapy was delivered. Nutritional fidelity was quantified as the proportion of prescribed calorie and protein targets achieved, this was required as an update to the original plan as supplements were not required by all patients.

**Statistical analysis**

In addition to descriptive analyses specified in the protocol, exploratory between-group comparisons were undertaken to inform the design and analysis plan of a future definitive trial. These analyses were not powered for effectiveness and are interpreted as hypothesis-generating.

**Health economic evaluation**

The protocol specified assessment of the feasibility of health economic data collection. The manuscript additionally reports preliminary resource use and cost estimates to further inform the design of a future economic evaluation.

**Intervention description**

Additional detail has been provided in the manuscript to improve reproducibility (including alignment with the TIDieR framework). These represent clarification of the intervention rather than modification.

**No changes**

No changes were made to study setting, eligibility criteria, randomisation procedures, intervention delivery, or outcome measures.

**Conclusion**

All amendments represent clarifications or extensions of reporting to support interpretation of feasibility outcomes and inform the design of a future definitive trial. No changes were made that would affect the internal validity of the study.
